# Identification and characterisation of microsatellite DNA markers in order to recognise the WSSV susceptible populations of marine giant black tiger shrimp, *Penaeus monodon*

**DOI:** 10.1186/s13567-015-0248-2

**Published:** 2015-09-25

**Authors:** Usri Chakrabarty, Sourav Dutta, Ajoy Mallik, Debabrata Mondal, Nripendranath Mandal

**Affiliations:** Division of Molecular Medicine, Bose Institute, P-1/12 CIT Scheme VII-M, Kolkata, 700054 India

## Abstract

White spot disease (WSD) which is caused by white spot syndrome virus (WSSV) creates severe epizootics in captured and cultured black tiger shrimp, resulting a huge loss in the economic output of the aquaculture industry worldwide. Performing selective breeding using DNA markers would prove to be a potential cost effective strategy for long term disease control in shrimps. In the present investigation, microsatellite DNA fingerprints were compared between naturally occurring WSSV resistant and susceptible populations of *Penaeus monodon*. After PCR with a set of shrimp specific primers three reproducible DNA fragments of varying sizes were found, among which 442 bp and 236 bp fragments were present in considerably higher frequencies in the WSSV susceptible shrimp population (*p* ≤ 0.0001). After WSSV challenge experiment the copy no. of WSSV was determined using real-time PCR, where it was found to be almost 4 × 10^3^ fold higher in WSSV susceptible shrimps than in the resistant ones. Thus, these microsatellite DNA markers will be useful to distinguish between WSSV susceptible and resistant brood stocks of *P. monodon.* Sequencing studies revealed that these DNA markers were novel in *P. monodon*. Highest WSSV resistance using these DNA markers, was observed in the shrimp populations of Andaman Island and Chennai among the different coastal areas of India, suggesting these places as safe for specific pathogen resistant brood stock shrimp collection. This study will be a very effective platform towards understanding the molecular pathogenesis of WSD for generation of disease free shrimp aquaculture industry.

## Introduction

Aquaculture industry enjoys an exponentially profitable market worldwide and deals with various valuable marine and fresh water invertebrate and vertebrate species. Marine black tiger shrimp, *Penaeus monodon*, is one of the most economically important native cultivated species of India due to its bigger size, higher nutritional value and huge demand as food delicacies across the globe. They contribute to 95% of the total production of the captured and cultured shrimps in India [1]. Proneness of the shrimp species towards many deadly viral diseases is an alarming factor in aquaculture industry [2]. Among them, the white spot disease (WSD) caused due to white spot syndrome virus (WSSV), a rod shaped dsDNA virus (genus *Whispovirus* of the family *Nimaviridae*) of 305.12 Kb length is the deadliest one. WSSV has a very wide host range and is able to sustain for a considerably long period of time in the virion state increasing the chances of further infection [3-5]. Several preventive measures were tested and applied throughout the world against the WSSV infection, but with a very less success rate [6-9].

It is well known that, some resistance phenomena always lies in the repository of the nature itself. Nature always favoured some individuals for better existance and resistance as well as preventive capability against any kind of odds like natural and artificial disasters. So that is obvious that, some kind of disease resistance phenomena might be present in shrimps also and some special genomic fingerprints may be accountable for this resistance.

Several DNA fingerprinting methods are used in population genetic studies, genetic diversity analysis, classifying germplasm and selective breeding in animals and plants for disease resistance [10-14]. Microsatellite markers are vastly used for study due to their reproducibility, co-dominant expression type, even genomic distribution, small locus size and high polymorphism. This enriched knowledge about DNA fingerprints can be very useful in the isolation of resistant individuals from an economically important species and cultivating them selectively as per the suitable genomic content. Disease resistance has been a major field of interest in several species of shrimps since decades. Eight markers associated with infectious hypodermal and hematopoietic necrosis virus (IHHNV) resistance or susceptibility in mutant and wild type populations of *Litopenaeus stylirostris* have been discovered using RAPD technique [15]. Evidence of clear association of a microsatellite marker was found with taura syndrome virus (TSV) susceptibility/resistance in *Litopenaeus vannamei* [16]. There are more instances where, one more microsatellite loci (*RS0622*) was identified to be associated with WSSV resistance in *Fenneropenaeus chinensis* [17]. Recently, single nucleotide polymorphism (SNP) discovery and association analysis detected several SNP markers associated with resistance to TSV in *L. vannamei* [18].

Previously, one 71 bp microsatellite DNA marker has been developed from two populations of *P. monodon* designated as WSSV resistant and disease susceptible which were collected from ponds, highly infected with WSSV [19]. Later both of these two populations of *P. monodon* were challenged individually by injecting WSSV and finally mortality as well as WSSV propagation was measured by quantitative real-time PCR. It was observed ~10^3^ fold higher WSSV propagation was occured in the disease susceptible population than the WSSV resistant population [20]. This study is mainly focused on the identification of more microsatellite DNA markers associated to WSSV resistance or susceptibility in shrimps. From the sequence analysis some very useful novel DNA markers are identified and practical applicability of these DNA markers are subsequently confirmed by WSSV challenge experiment. The possible safer places for WSSV resistant brood stock collection for Indian shrimp aquaculture are also suggested in this investigation.

## Materials and methods

### Sample collection

*P. monodon* samples were collected from 20 highly disease affected ponds in West Bengal, India. The mature adult shrimp samples of 75 days post larva weighed ~55 g in average were collected from the culture ponds where the water temperature was 25°-30 °C and the infection lasted for 3–4 days. The collected shrimps were divided into two groups, WSSV resistant (*n =* 195) and disease susceptible (*n* = 255). The former population survived well in the affected ponds, had no clinical signs of disease and were found to be WSSV negative by nested PCR; the latter population, however, died because of disease with obvious clinical signs and were observed WSSV positive by PCR.

### WSSV screening by nested PCR

WSSV was qualitatively tested using genomic DNA from gill tissue of shrimps according to the method reported previously using commercially available kit (GeNei™, MERCK, India) [21]. The gel photograph was documented in a gel documentation system (EC3 Chemi HR 410 Imaging System, UVP, USA).

### Genomic DNA extraction

The pleopod tissues (~100 mg) were collected from individual shrimps and subjected for genomic DNA preparation using phenol-chloroform method [22]. Each DNA sample was quantified by UV spectrophotometer (Shimadzu UV160U, Japan) and electrophoresed in 0.8% agarose gel to determine their quality before using in subsequent PCR reactions.

### Identification of disease susceptible and WSSV resistant population of *P. monodon* using microsatellite DNA markers

To accomplish the PCR reaction from the isolated shrimp genomic DNA, shrimp specific primers (forward: OM 73 and reverse: OM 74) [23] were taken from the microsatellite locus (PM205; Accession no - AY500854) of *P. monodon*. The PCR reaction for the microsatellite amplification was carried out in a 25 μL reaction mixture containing 400 ng genomic DNA, 30 pmol forward and reverse primer (Biotech Desk, India), 0.2 mM dNTPs (MP Biomedicals, USA), 1 mM MgCl_2_ (MP Biomedicals, USA), 1X buffer (10 mM Tris–HCl, pH 9.0, 50 mM KCl, 0.1% Triton X100, 0.2 mg mL^−1^ BSA) (MP Biomedicals, USA) and 1.0 U *Taq* DNA polymerase (MP Biomedicals, USA). Each reaction mixture was placed in a thermal cycler (PTC100, MJ Research Inc., USA). The thermal profiles for PCR was as follows: 94 °C for 5 min; followed by 35 cycles of 94 °C for 45 s, experimental annealing temperature at 50–60 °C for 1 min and extension at 72 °C for 1 min, after that final extension at 72 °C for 5 min. The amplified DNA fragments were separated by electrophoresis in 2.5% agarose gel at 80–90 volt for 2–3 h and subsequently visualized by staining with ethidium bromide (1 μg mL^−1^). WSSV was extracted using PEG virus precipitation kit from WSSV infected tissue (hepatopancreas and gill) of shrimp (BioVision, USA). DNA was extracted from WSSV using High Pure Viral Nucleic Acid Kit (Roche, Germany) and was subjected to PCR with microsatellite primers following above mentioned protocol to confirm the shrimp origin of these DNA markers. This method was also applied later for the samples of challenge experiment after preparation of genomic DNA by phenol-chloroform method.

### WSSV challenge test to confirm the significant association between DNA markers and disease susceptibility

Live *P. monodon* samples (~50 g body weight, *n* = 382), devoid of any WSSV infection, were collected from ponds and kept for acclimatization before the challenge for two days in recirculatory marine aquarium at 23–26 °C temperature and 6–8 gl^−1^ salinity. Some essential salts were added and the marine aquarium system was standardized to provide optimal conditions in favor of growth and molting of shrimps. For maintaining appropriate healthy natural aquatic environment some chemicals that are widely used in aquaculture farms, viz., PondDtox® and PondProtect® (Novozyme, Europe) were also used in the aquarium. For the challenge experiment WSSV stock solution was prepared using PEG virus precipitation kit from ~48 g of WSSV infected tissue (hepatopancreas and gill) of shrimp (BioVision, USA). The bioassay experiment was performed to determine the virus titer responsible for 50% mortality, which was found at 10^4^ dilution of virus stock. Therefore, 40 μL of 10^5^ dilution of virus solution was injected into the tail muscle of each shrimp (*n* = 240) and genomic DNA was extracted from tail tissue of shrimps after 72 h of WSSV challenge experiment. Survivability as well as WSSV propagation by real-time PCR was also recorded at post 72 h.

### WSSV quantification for the samples of WSSV challenge experiment by real-time PCR

Quantification of WSSV copies in shrimp samples were determined by comparing the average C_T_ (cycle threshold) values with the standard curve by real-time PCR prepared by plotting various C_T_ values against different consecutive dilutions of standard plasmids. The real-time PCR reaction was carried out in a thermal cycler (StepOnePlus™, Applied Biosystems, USA) with 10 μL reaction mixture containing 10 ng genomic DNA from pleopod tissue, 3 pmol of each WSSV specific primers producing 50 bp amplicon (Biotech desk, India) and 0.8X SYBR green master mix (Applied Biosystems, USA). The thermal profile was, 50 °C for 10 min, 95 °C for 10 min followed by 40 cycles of 95 °C for 15 s, 50–55 °C for 30 s, 72 °C for 45 s. All the samples for real-time PCR were run in duplicates and *t-* test was performed to observe the mean C_T_ value among the challenged samples.

### Cloning and sequencing of microsatellite DNA markers

The 442 bp and 236 bp microsatellite DNA markers were cloned and sequenced by using an ABI PRISM dye terminator ready reaction kit followed by the manufacturer protocol (Applied Biosystems, USA). The sequence was analysed in the NCBI (USA), EMBL (Europe) and DDBJ (Japan) nucleotide Blast program for homology searching with known sequence database.

### Determination of disease susceptible and disease resistant population of *P. monodon* using microsatellite DNA markers along the entire coastal areas of India

Almost 100 shrimp samples (~100 gm each) were collected from 9 different coastal areas along Indian coastline. East coast samples were collected from Digha, West Bengal (co-ordinates: 21°38′N, 87°33′E); Chilika, Orissa (co-ordinates: 20°1′N, 85°32′E); Visakhapatnam, Andhra Pradesh (co-ordinates: 17°41′N, 83°18′E); Chennai, Tamil Nadu (co-ordinates: 13°02′N, 80°10′E), Port Blair, Andaman (co-ordinates: 12°16′N, 93°51′E). West coast samples, on the other hand, were collected from Kochi, Kerala (co-ordinates: 9°96′N, 76°21′E); Mangalore, Karnataka (co-ordinates: 12°87ʹN, 74°88ʹE); Vasco-Da-Gama, Goa (co-ordinates: 15°40ʹN, 73°83ʹE); and Veraval, Gujarat (co-ordinates: 20°90ʹN, 70°37ʹE). The PCR was accomplished following protocol described above during microsatellite DNA marker identification to identify disease susceptible or resistant shrimp population from the isolated shrimp genomic DNA collected from different coastal areas.

### Statistical analysis

The molecular size of the amplified PCR products were calculated using DNA markers by Molecular Analyst software (VisionWorksLS, UVP, USA). The polymorphism of DNA bands were carefully analyzed among WSSV resistant and WSSV susceptible shrimp population and the significance level of frequency of band (presence and absence in two populations) was calculated by Fisher’s exact test. The mean mortality assay between WSSV resistant and WSSV susceptible individuals was analyzed using the statistical program (SPSS v10.0 and MS Excel 2010). The real-time PCR data were analyzed by StepOne software v2.1 (Applied Biosystems, USA). The *t-* test was performed to differenciate between the mean C_T_ value of the WSSV resistant and disease susceptible samples by statistical software Kyplot (version 2.0 beta 13). The cor-relation analysis between the two developed microsatellite DNA markers was performed by a statistical program (SPSS v10.0 and MS Excel 2010).

## Results

### Detection of WSSV by nested PCR in collected natural shrimp population

**Figure 1:**
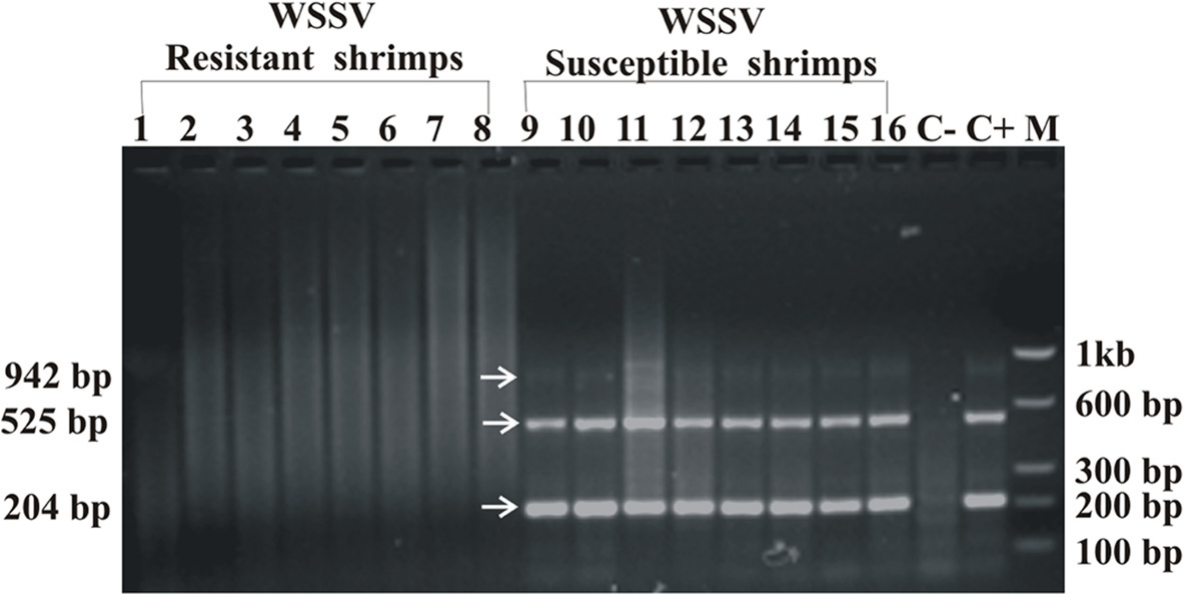
**WSSV detection test by commercially available kit.** The PCR amplified DNA was electrophoresed in 2% agarose gel. Lanes 1–8 showed the no amplification of DNA bands specific to WSSV from WSSV resistant *Penaeus monodon.* Lanes 9–16 showed WSSV specific amplified DNA bands of 942 bp, 525 bp and 204 bp from disease susceptible *P. monodon.* Lane C-, a negative control with no viral DNA. Lane C+, a positive control produced all the three fragments (942 bp, 525 bp and 204 bp) that indicated very high concentration of virus (≥10^5^ viral particle). Lane M indicated the molecular weight marker. The molecular weights of the bands specific to the different copies of WSSV are indicated by the arrows at the left hand side of the gel in the figure.

WSSV specific primers using commercially available kit (GeNei™, MERCK, India) were used for PCR and generated three DNA fragments of 942 bp, 525 bp and 204 bp respectively from WSSV genome based on the severity of infection. Very high infection (≥10^5^ viral particles) produced all the three DNA fragments (942 bp, 525 bp and 204 bp), whereas moderate to high infection (≥10^3^ - <10^5^ viral particles) produced two DNA fragments (525 bp and 204 bp) and low infection (10 to 200 viral particles) produced only the 204 bp DNA fragment. Lanes 1–8 in Figure 1 represents the absence of WSSV in the gill tissues of the individuals of WSSV resistant population in single tube nested PCR by WSSV-specific primers provided with the kit. However, moderate to high WSSV infection (with 10^3^-10^5^ WSSV particles) was observed in the gill tissues of the disease susceptible individuals, as reflected in lanes 9–16 in Figure 1. Among the collected shrimp overall 56.66% of individuals were found to be naturally infected by WSSV while 43.33% individuals were found devoid of virus at the time of their capture.

### Microsatellite DNA marker analysis of shrimp genomic DNA

**Figure 2:**
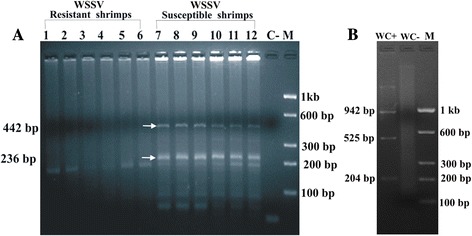
**Microsatellite DNA marker analysis of shrimp and WSSV genomic DNA.** Genomic DNA was isolated from pleopod tissue of *Penaeus monodon* and subjected to microsatellite DNA marker analysis. PCR amplified DNA fragments were electrophoresed in 2.5% agarose gel and photographed after staining with ethidium bromide. In panel **A** Lanes 1–6 show PCR amplified DNA bands from WSSV resistant *P. monodon*. Lanes 7–12 show PCR amplified DNA bands from disease susceptible *P. monodon*. Lane C- is the negative control and Lane M indicates molecular weight marker. Number in the right side indicated the molecular size of molecular weight marker and at the left hand side the molecular weights of the significant bands specific to the susceptible samples are indicated by the arrows in the figure. In panel **B** WC+ shows the amplified DNA bands in the nested PCR of WSSV genomic DNA with WSSV specific primers, WC- shows no amplified DNA bands in the PCR of WSSV genomic DNA with shrimp specific microsatellite primers (OM 73 and OM 74).

PCR amplification by the shrimp specific primers taken from microsatellite locus of *P. monodon* produced two significant DNA fingerprints in order to differentiate between WSSV resistant and disease susceptible shrimp populations. Upon PCR amplification two bands of molecular sizes 442 bp and 236 bp were generated in disease susceptible population whereas, absence of these two bands were evident in the WSSV resistant population. Moreover, appearance of another 215 bp DNA band was observed in both the populations (Figure 2A). These results illustrate two reproducible microsatellite DNA markers in disease susceptible population. Table 1 shows the allele frequencies of 442 bp were 0.39 and 0.43 in WSSV resistant and susceptible populations while in case of 236 bp the frequency in WSSV resistant and susceptible populations were 0.21 and 0.27, respectively. *Chi* square was calculated with these data, and value was optimised by applying Yates’ correction using Fischer's Exact Test. The 442 bp and 236 bp fragments are highly statistically significant (*p* ≤ 0.0001) microsatellite DNA markers which can distinguish between WSSV resistant and susceptible populations of *P. monodon*.

**Table 1:** **Microsatellite markers associated to disease susceptibility in** ***Penaeus monodon*** **: their frequencies and statistical significances**

**Location**	**WSSV Resistant (** ***n*** **= 195)**			**WSSV Susceptible (** ***n*** **= 255)**			***Chi*** **-square Value**	***p*** **value**	**Significance**
Twenty various WSSV affected ponds	Allele(bp)	# of obs	Frequency^a^	Allele(bp)	# of obs	Frequency^a^			
	442	74	0.39	442	149	0.43	18.139	**<**0.0001***	HS
	236	40	0.21	236	93	0.27	13.281	**<**0.0001***	HS
	215	73	0.39	215	102	0.30	0.260	0.626	NS
Total	3	187	-	3	344	-	23.480	<0.0001***	HS

*Chi* square value was calculated and yates’ correction was applied, while the probability was calculated by using fischer's exact test.

*n*: number of samples for each group.

NS: not significant; ***(*p* ≤ 0.001): HS/highly significant.

^a^Frequency of allele = number of observation at the given allele/total number of observation of all alleles in a particular population (either in WSSV resistant or WSSV susceptible population).

PCR amplification with shrimp specific primers (OM 73 and OM 74) from viral genomic DNA did not produce any DNA band, whereas PCR amplification using virus specific primers only produced appropriate DNA bands (Figure 2B).

### Mortality analysis among the WSSV resistant and disease susceptible shrimps after WSSV challenge experiment

**Figure 3:**
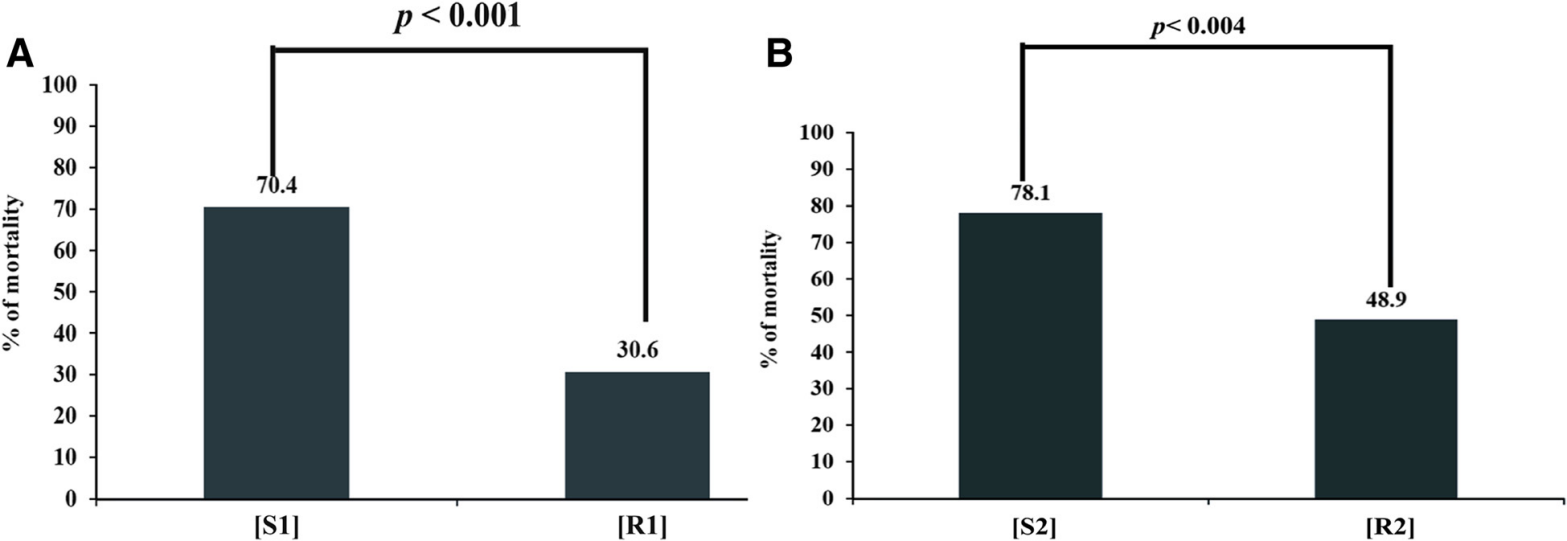
**Mortality analysis by 442 bp and 236 bp microsatellite DNA marker after WSSV Challenge experiment.** Mortality ratio between disease resistant and susceptible population is statistically significant (*p* < 0.001 and *p* < 0.004). In panels **A** and **B**, R1 and R2 signifies WSSV resistant population based on 442 bp and 236 bp microsatellite DNA marker, respectively and S1 and S2 denoted Susceptible population based on 442 bp and 236 bp microsatellite DNA marker respectively.

Among the total of 382 individuals of challenged *P. monodon*, 240 samples were found WSSV negative prior to the challenge and were therefore taken for subsequent result analysis. The percentages of mortality among disease susceptible shrimps were 70.4% (*p* < 0.001) and 78.1% (*p* < 0.004), according to the 442 bp and 236 bp DNA markers, respectively (Figures 3A and B). While in case of resistant population, percentages of mortality were 30.6% and 48.9%, respectively for 442 bp and 236 bp markers. The percentage of mortality of WSSV resistant population was significantly different (*p* < 0.001) from the WSSV susceptible population.

### Quantification of WSSV among WSSV resistant and WSSV susceptible shrimps after WSSV challenge experiment

**Figure 4:**
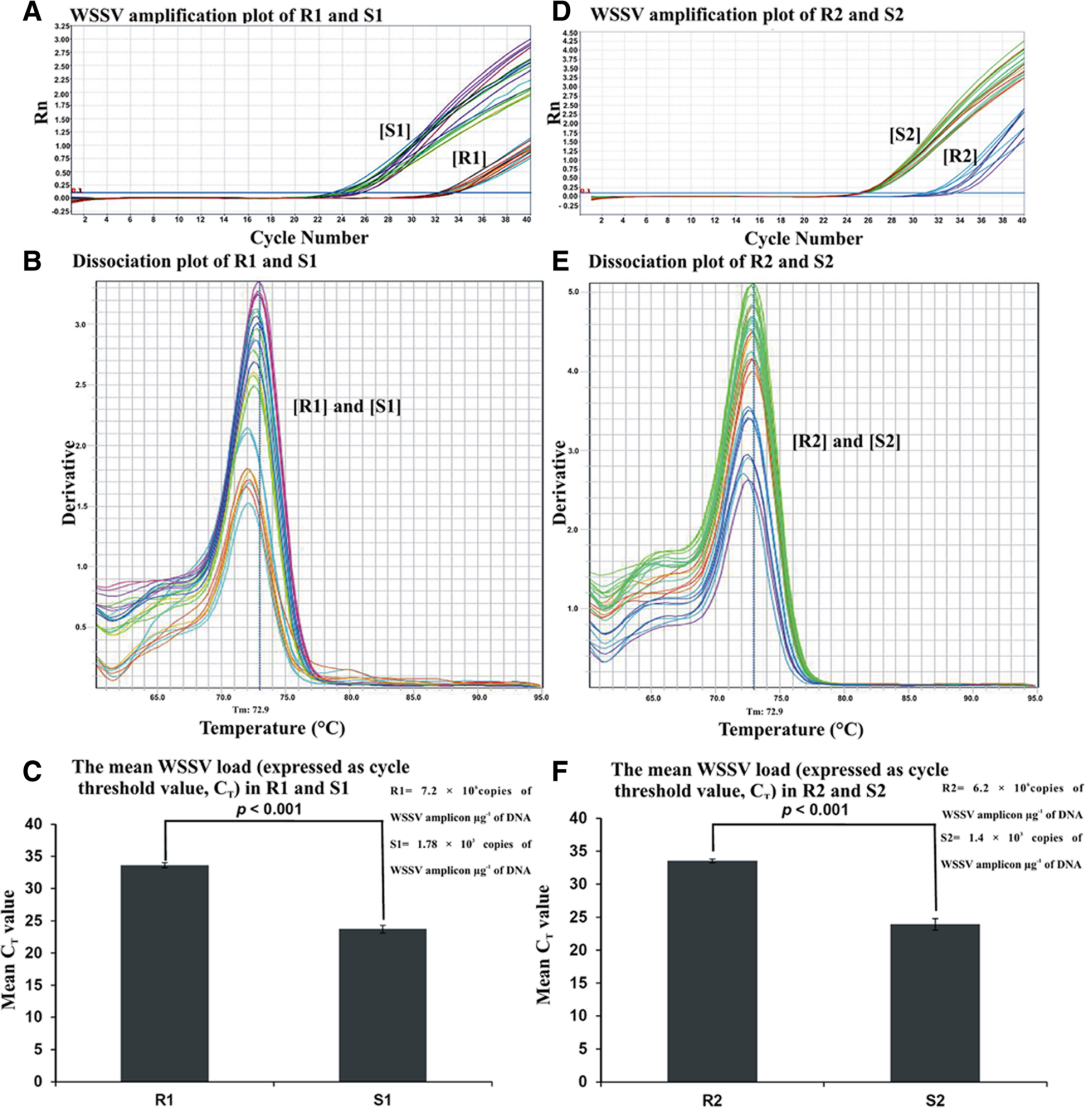
**Quantitative assay of WSSV after experimental virus challenge.** The amplification plots (**A** and **D**) of white spot syndrome virus (WSSV) in WSSV resistant [R1 = samples devoid of 442 bp band and R2 = samples devoid of 236 bp band] and susceptible populations [S1 = samples containing 442 bp band and S2 = samples containing 236 bp band] of *Penaeus monodon* based on 442 bp and 236 bp microsatellite DNA marker respectively. Genomic DNA from tail tissue was amplified using WSSV (**A** and **D**) primers by real-time PCR after 72 h challenge experiment. The mean relative WSSV was expressed as cycle threshold value, C_T_ between [R1 and R2] and [S1 and S2] populations of *P. monodon*. A two sample *t*-test showed highly statistically significant difference between these two populations (**C** and **F**, *n* = 240, *P* < 0.001) using both the microsatellite DNA marker. The dissociation curves for WSSV amplicon are shown in panels (**B**) and (**E**) for two populations ([R1 and R2] and [S1 and S2]) differentiated by 442 bp and 236 bp microsatellite DNA marker. The T_m_ value indicated the same WSSV amplicon in every case.

WSSV quantification was performed by comparing the C_T_ value of the experimental samples with the standard curve, which was obtained by using different consecutive dilutions of standard plasmids. Figure 4 showed the amplification plots of WSSV using real-time PCR among WSSV resistant and WSSV susceptible populations of *P. monodon* based on 442 bp (Figure 4A) and 236 bp (Figure 4D) DNA markers. The dissociation curves showed amplification of the similar amplicons of WSSV in two different populations according to both 442 bp and 236 bp microsatellite markers (Figures 4B and E). Results indicated that the mean C_T_ ± SEM values in WSSV resistant and WSSV susceptible shrimps based on 442 bp microsatellite DNA marker were 33.6 ± 0.4 and 23.7 ± 0.59 whereas, the same were 33.5 ± 0.3 and 23.9 ± 0.87 in two populations discriminated using 236 bp microsatellite DNA marker. The difference in the mean C_T_ values of WSSV amplicons were highly statistically significant (Figures 4C and 4F; *p* < 0.001) among WSSV resistant and susceptible populations designated by both markers. The viral load was determined from standard curve and it was found that the WSSV susceptible population had more than 10^3^ fold higher WSSV amplicon in μg^−1^ of DNA than the resistant population (Table 2). Whereas, beta actin amplicon showed no significant difference in C_T_ value (± SEM) of WSSV resistant (22.9 ± 0.7) and susceptible samples (22.2 ± 1.03) distinguished by both markers.

**Table 2:** **The absolute WSSV copy number at 72 hours post WSSV challenge**

**Sl No**	**DNA Marker**	**Mean copy number μg** ^**−1**^ **of total extracted DNA at 72 hours post challenge**		***p*** **-Value**	**Significance**
		**WSSV Susceptible shrimps**	**WSSV Resistant shrimps**		
1	442 bp	7.2 × 10^6^	1.78 × 10^3^	**<**0.001**	HS
2	236 bp	6.2 × 10^6^	1.4 × 10^3^	**<**0.001***	HS

***(*p* ≤ 0.001): HS/highly significant.

### Sequences of WSSV susceptible DNA markers

The 442 bp and 236 bp microsatellite DNA markers were sequenced. Subsequently, NCBI BLAST programme was performed but no such significant similarity was found with the known database. This firmly indicates towards the novelty of these sequences and their specificity to black tiger shrimp. These sequences were submitted to NCBI nucleotide database under the following GenBank IDs: KP751417 and KP751418.

### WSSV resistant prevalence among the collected shrimps using 442 bp and 236 bp microsatellite DNA marker along the entire coast of India

**Figure 5:**
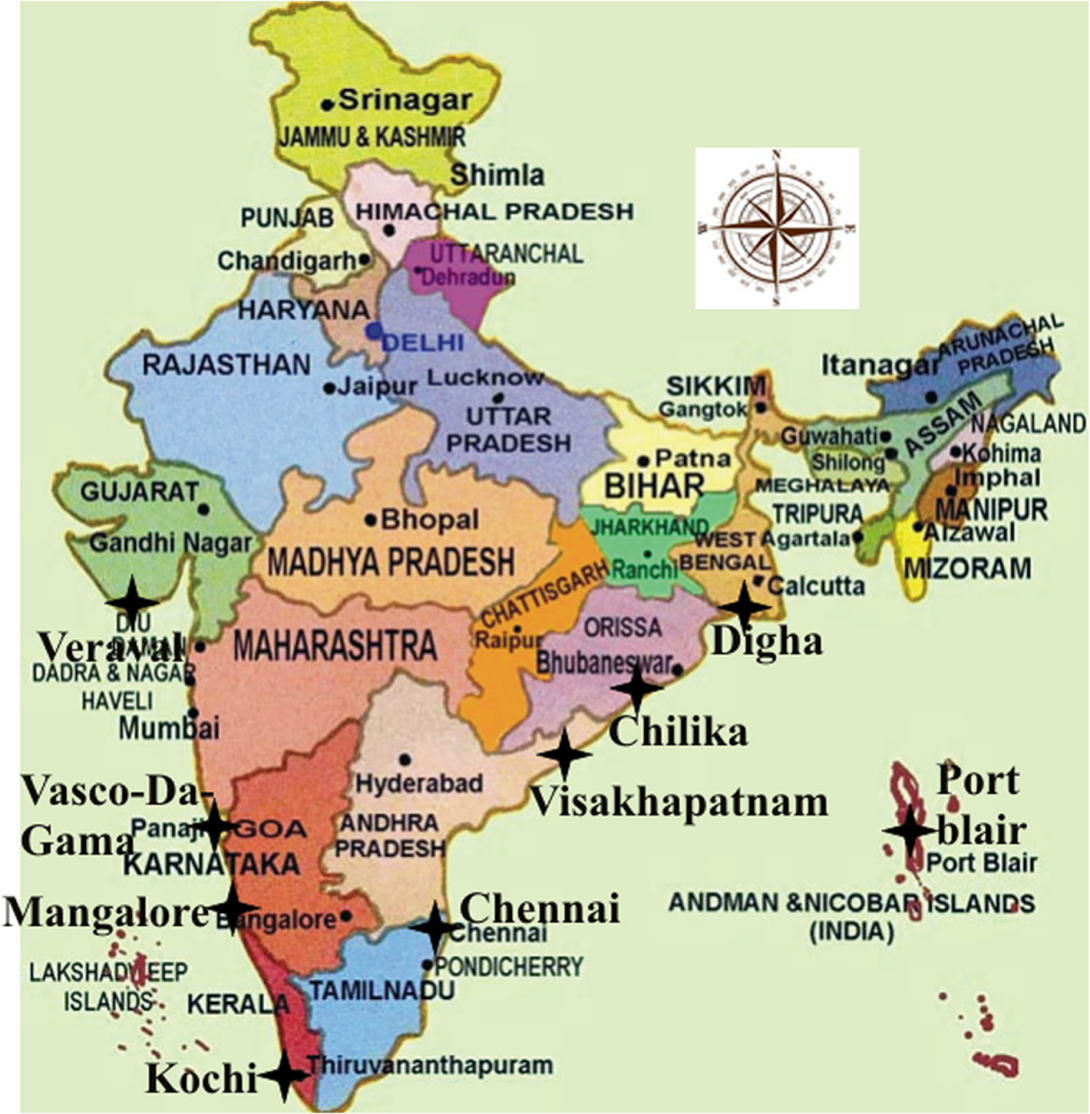
**India Map with studied area location mark up.** The nine geographic locations along the entire coastline of India are marked with black stars (). In the East coast samples were collected from Digha, West Bengal; Chilika, Orissa; Visakhapatnam, Andhra Pradesh; Chennai, Tamil Nadu and Port blair, Andaman while in the West coast samples were collected from Kochi, Kerala; Mangalore, Karnataka; Vasco-Da-Gama, Goa and Veraval, Gujarat.

**Figure 6:**
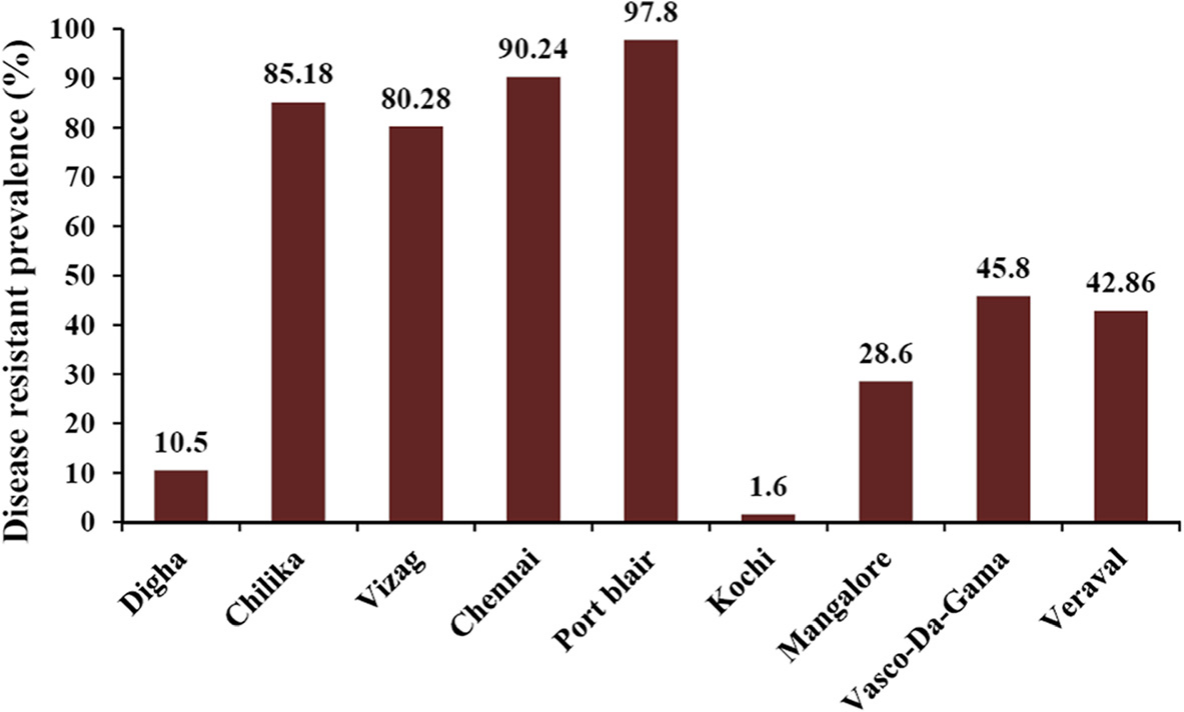
**WSSV resistant prevalence along the entire coastal areas of India.** Mean WSSV resistant prevalence using 442 bp and 236 bp microsatellite DNA marker among the wild population of *Penaeus monodon*.

The shrimp samples which appeared negative for WSSV after nested PCR were considered for WSSV resistant prevalence calculation. Samples where the microsatellite markers were absent, were considered as WSSV resistant. The mean WSSV resistant prevalence using both DNA markers showed 10.5%, 85.18%, 80.28%, 90.24%, 97.8%, 1.56%, 28.57%, 45.83% and 42.86% of the samples from Digha, West Bengal; Chilika, Orissa; Visakhapatnam, Andhra Pradesh and Chennai, Tamil Nadu, Port blair, Andaman; Kochi, Kerala; Mangalore, Karnataka; Vasco-Da-Gama, Goa; Veraval, Gujarat, respectively (Figure 5). As it can be seen, WSSV resistant prevalence was highest in Port Blair from the East coast and in Vasco-Da-Gama from the West coast, among the coastal areas of India (Figure 6).

### Correlation analysis in between the two microsatellite DNA markers

**Figure 7:**
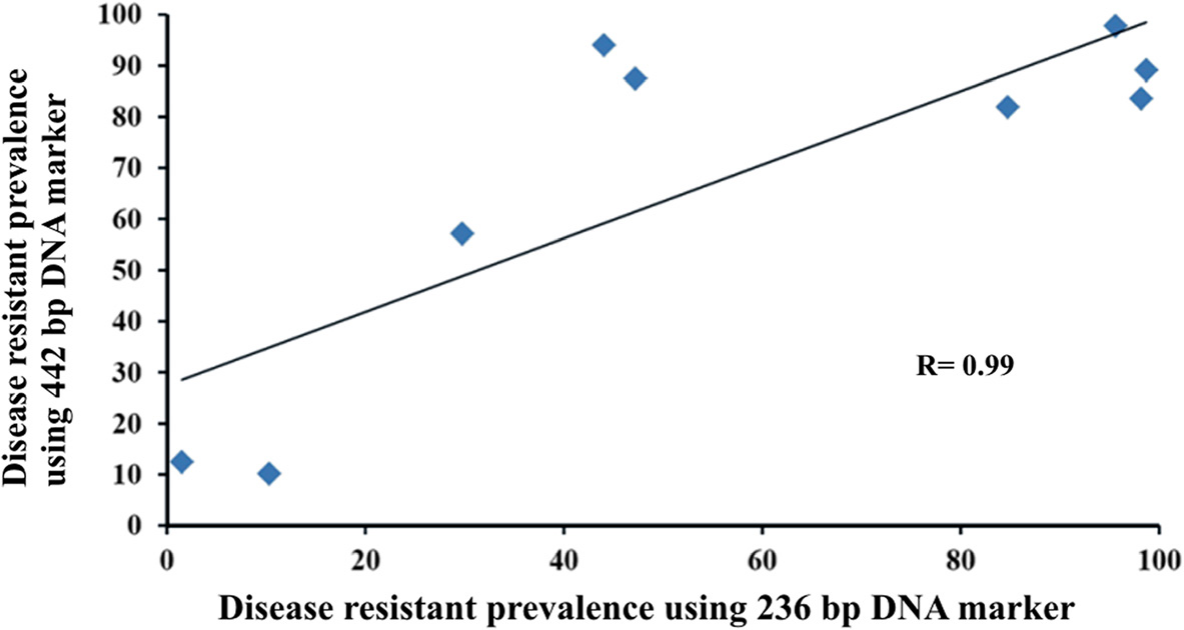
**Mean WSSV resistant prevalence using developed 442 bp and 236 bp microsatellite DNA markers.** The mean WSSV resistant prevalence along the East and West coast of India is positively correlated among each other.

The mean WSSV resistant prevalence using developed 442 bp and 236 bp microsatellite DNA markers along the East and West coasts of India is positively correlated with the result of 236 bp microsatellite DNA marker (Figure 7). This strong positive correlation may indicate that these markers belong to similar Quantitative Trait Loci (QTL).

## Discussion

White spot disease of shrimps is a highly contagious water borne infection, that has the ability to eradicate an entire shrimp population, within a very short period of time. By virtue of its brilliant adaptivity, WSSV persists in the cultured ponds even after they are completely dried at the end of the cultivation season. The vertical and horizontal transmissions of this lethal virus may occur from infected broodstock to their offsprings, through cannibalism of moribund shrimps and even through water exchange between nearby farms [24]. Various oral vaccination experiments to immunize the shrimp samples by viral envelope proteins and bacterial strains of *Vibrio* have been previously tested [6,25]. The lack of adaptive immunity in the invertebrate system and innate immunity being the sole line of defence, it usually results a decrease in the vaccine induced resistance against viruses after a few days. Moreover, this exertion also has many limitations as to be applied practically in a large scale field. To generate improved black tiger shrimp breeding lines with higher survival rate, greater production in terms of feed to biomass conversion potency and reduced recurring cost can only be done by understanding the shrimp genome constituent and inheritance mode using DNA markers [26]. Different research groups were involved in the development of WSSV resistant broodstock of *P. vannamei* by WSSV challenge experiment [27]. Similarly, based on the clinical symptoms, the existence of WSSV resistance or tolerance in *P. monodon* following WSSV challenge experiment has been reported [28]. Studying the quantitative genetics of WSSV resistance in *P. vannamei* revealed that WSSV resistance is a polygenic trait with strong individual effluences in *P. vannamei* [29].

Previously, a 71 bp microsatellite DNA marker in *P. monodon* linked to WSSV susceptibility has been reported [19]; after WSSV challenge greater viral copies were detected by quantitave real-time PCR in WSSV susceptible population of shrimp [20]. In this present investigation, new microsatellite DNA markers that may be useful in identifying *P. monodon* population associated to WSSV susceptibility were detected. It has been observed that a highly significant difference exists in the microsatellite fingerprints of WSSV resistant and susceptible populations of *P. monodon*. The susceptible population generated two additional DNA fragments of 442 bp and 236 bp which were mostly absent in the WSSV resistant population. Whereas, another fragment of 215 bp appeared that was omnipresent for both the populations. Absence of product formation after amplification with shrimp specific primers and WSSV DNA clearly suggested that, these microsatellite DNA markers were specific to *P. monodon*. There was no sequence homology in between these DNA markers and the already reported complete WSSV genome at NCBI/EMBL/DDBJ genbank.

The main theme of this study was to describe the natural potential for WSSV resistance in shrimp system by studying its DNA content polymorphisms and making it implicable in aquaculture practice. For this purpose, two populations of *P. monodon* were collected from ponds highly infected with WSD where almost 90-99% of the shrimps died after the advent of WSSV infection but only 1-10% survived in the same affected pond at the same time [30]. These ponds were seeded with a random collection of post larva obtained from the wild marine brooders having variable genomic contents. After development of the markers with this WSSV infected shrimps from culture ponds, pathogen free live shrimps were again collected to perform a laboratory WSSV challenge experiment in order to strengthen the aforementioned hypothesis. For this purpose, shrimp samples were selected by 442 and 236 microsatellite markers prior to the WSSV injection, after which mortality data was recorded at 72 h. Post challenge data showed that the rate of mortality among WSSV susceptible shrimps were significantly higher than the WSSV resistant shrimps. Quantitative real-time PCR data also strengthened this observation by showing 4.04 × 10^3^ and 4.4 × 10^3^ fold higher virus copies in disease susceptible shrimps at 72 h of challenge experiment than that of resistant samples. The sequence homology searching of these microsatellite DNA marker using NCBI/ EMBL/ DDBJ blast programme was performed. It was found that these DNA markers are novel and unique to shrimps only. This study also suggests the possible safer places for WSSV resistant seed collection in India. At the East coast, Chennai and Port Blair can be good places for collection of gravid female for further seed generation from it for sustainable WSSV resistant aquaculture. Vasco-Da-Gama, Goa can also be a good option for WSSV resistant seed collection at the West coast of India. The strong positive correlation between two microsatellite DNA markers indicates these markers can be regulated by similar mechanism and may be present in same QTL.

In conclusion, this study is a foundation for further characterization of the disease related microsatellite DNA markers in *P. monodon*. Although, it is important to develop more WSSV resistant DNA markers associated with QTL(s), that probably will give a clearer idea about the polygenic status of the WSSV resistance trait in *P. monodon*. Additionally, it will be important to find out the role of these markers in the WSSV resistant population. At present it is not clear whether this marker specific to *P. monodon* are indicative of a WSSV resistant QTL region or if so, then the molecular pathogenesis will be a very good field of study. This observation also suggested that this marker would be useful for the generation of successful SPR breeding program through marker assisted selection (MAS) to give a new lease of life to the aquaculture industry.
